# Ossification of ligamentum flavum related to progressive thoracic myelopathy and acute paraplegia in a Central-European male with a thoracic kyphoscoliosis

**DOI:** 10.1093/jscr/rjad070

**Published:** 2023-02-23

**Authors:** Danijel Erdani, Matevž Topolovec, Nikša Hero, Peter Brumat

**Affiliations:** Department of Spine Surgery, Valdoltra Orthopaedic Hospital, Jadranska cesta 31, 6280 Ankaran, Slovenia; Faculty of Medicine, University of Ljubljana, Vrazov trg 2, 1000 Ljubljana, Slovenia; Department of Spine Surgery, Valdoltra Orthopaedic Hospital, Jadranska cesta 31, 6280 Ankaran, Slovenia; Faculty of Medicine, University of Maribor, Taborska ulica 8, 2000 Maribor, Slovenia; Department of Spine Surgery, Valdoltra Orthopaedic Hospital, Jadranska cesta 31, 6280 Ankaran, Slovenia; Department of Spine Surgery, Valdoltra Orthopaedic Hospital, Jadranska cesta 31, 6280 Ankaran, Slovenia; Faculty of Medicine, University of Ljubljana, Vrazov trg 2, 1000 Ljubljana, Slovenia

## Abstract

The ossification of the ligamentum flavum (OLF) presents a significant risk factor in the development of spinal cord compression in the (lower) thoracic spine, particularly in eastern Asian elderly males. The definite causes for OLF have not yet been fully uncovered, whereby age, genetics, metabolic disorders and mechanical stress are deemed among the most plausible pathophysiological factors in OLF. Spinal deformities (mostly kyphotic) are associated with an excess in tensile forces, which may lead to hypertrophy and OLF. This unique case of OLF-related acute paraplegia and progressive thoracic myelopathy in a Central-European male patient may indicate the role of a (kyphoscoliotic) spinal deformity in the initiation and progression of the OLF-related (thoracic) myelopathy. Promptly initiated surgical decompression and (partial) deformity correction may, along with proper subsequent intradisciplinary rehabilitation process, greatly improve the clinical outcome post-treatment, especially in terms of quality of life and residual pain.

## INTRODUCTION

Despite being a relatively rare pathology, the ossification of the ligamentum flavum (OLF) has been recognised as a significant cause of spinal stenosis and its associated neurologic disturbances, particularly in the thoracic part of the spinal canal [1, 2]. The prevalence of the OLF is the highest in the thoracic spine, especially in elderly male patients in Asia, whereby the ossification of other spinal ligaments can also overlap with the OLF [1–3]. Although various factors are linked to the pathophysiologic development of OLF, its etiology remains uncertain [4, 5].

Spinal deformities, such as thoracic kyphosis, have also been associated with the development of OLF due to disturbed biomechanics of the affected area and subsequent degeneration of the involved segments [1, 2, 4, 6]. Posterior laminectomy is the standard of care for symptomatic, OLF-associated spinal cord compression, despite the considerable perioperative complications [2, 5].

## CASE REPORT

A 41-year-old Central-European male was referred to our institution for emergent surgical treatment due to acute aggravation of progressive thoracic myelopathy with acute paraplegia and painful concomitant thoracic kyphoscoliosis. The patient reported numbness of both lower limbs and the perineum, with signs of a hyperreactive neurogenic bladder. Since perinatal brain injury, he had residual spastic tetra-paresis. In his childhood, the patient was diagnosed with thoracic kyphoscoliosis, which did not require correction at the time.

Eleven years prior to the referral to our institution, the patient had been operated on in an outside facility for an intramedullary cavernous hemangioma at the level of Th2 via Th1-Th3 laminectomy, myelotomy and excision, followed by subsequent revision surgery with the extension of laminectomy to C7, due to a relapse 4 years after the index surgery. Since the second surgery, the spasticity and hypoesthesia of the patient’s entire left lower limb remained unchanged. Despite the residual neurological deficit and being wheelchair-bound for longer distances, the patient was able to ambulate shorter distances with crutches and drive a modified car. The residual back pain was manageable with conservative measures.

**Figure 1:**
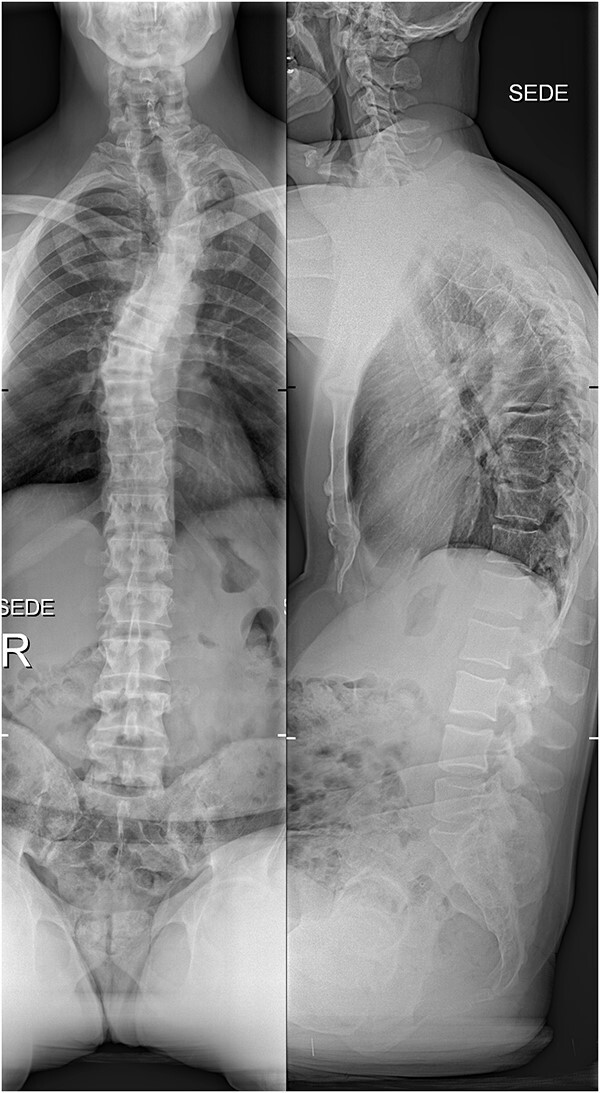
The initial X-ray images of the thoracic spine at the time of admission to our institution. Anteroposterior view (left) and lateral view (right).

**Figure 2:**
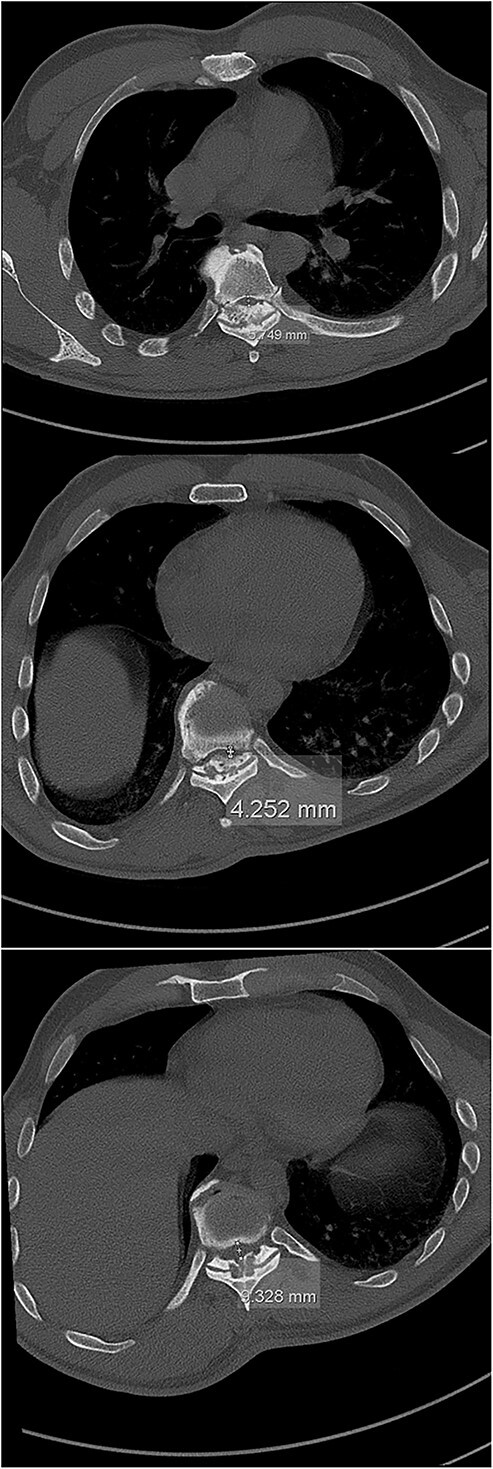
The initial CT scan (axial views), showing the width of the spinal canal at Th6 (above), Th9 (middle) and Th11 (below).

**Figure 3:**
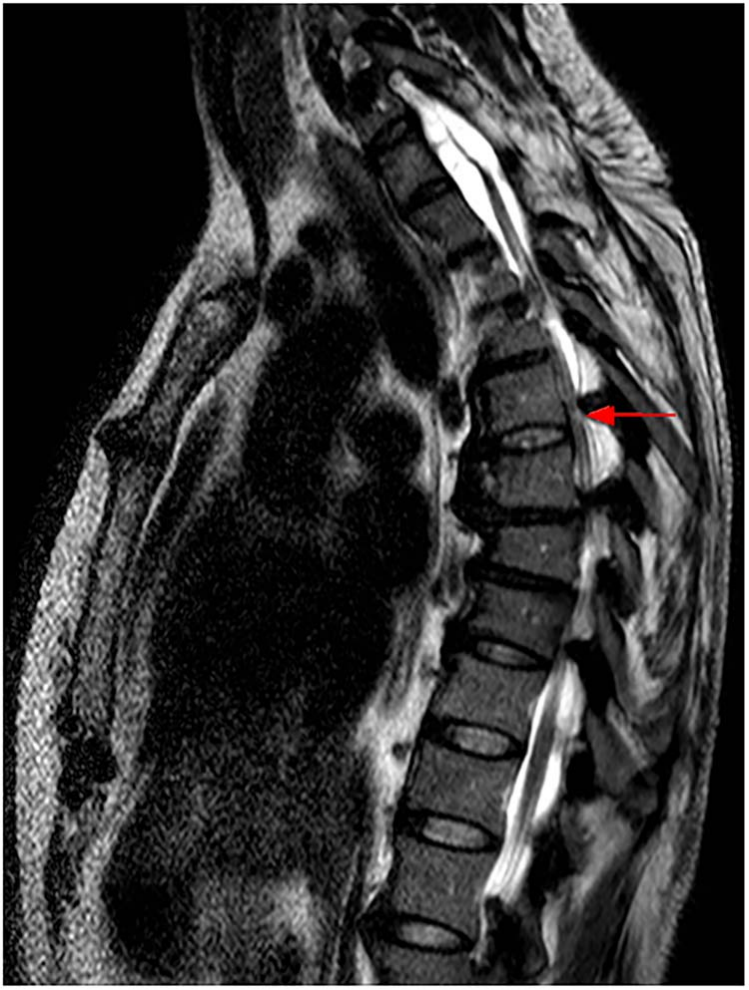
MRI scan of the thoracic spine at the time of admission, with hyperintense intramedullary T2 signal at Th6, indicating myelopathy (red arrow).

The imaging studies revealed thoracic kyphoscoliosis (with a kyphotic curve of 50°, left upper-thoracic curve of 40° and right lower-thoracic curve of 25°) and significant stenosis of the thoracic part of the spinal canal (particularly at levels Th6, Th9 and Th11) due to severely ankylosed ligamentum flavum (OLF) and the concomitant degeneration of the facets (Figs 1 and 2). Imaging indicated thoracic myelopathy (Fig. 3). We performed emergent posterior wide laminectomy using an ultrasound bone scalpel, and a partial correction of the deformity by instrumented spinal fusion (Th6-Th12). The surgery was performed by the senior author (N.H.).

**Figure 4:**
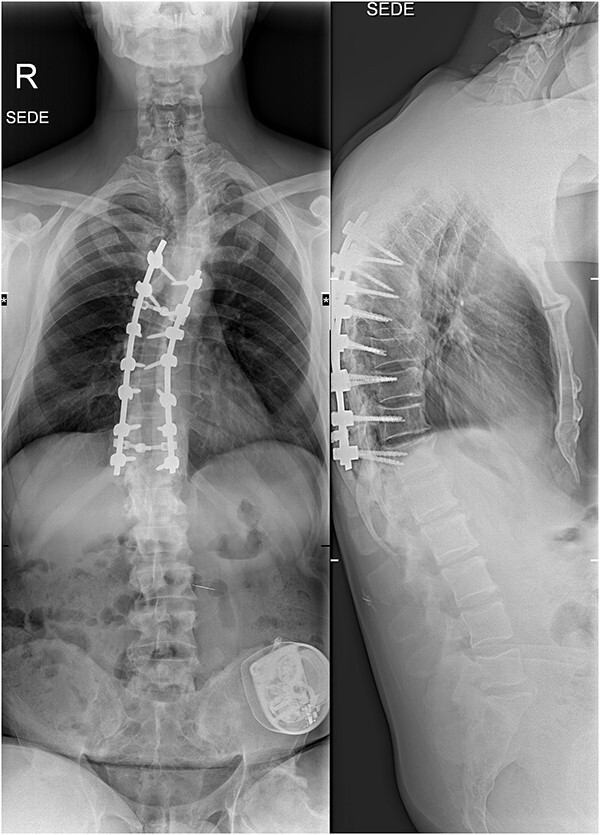
X-rays at the final follow-up 4 years after the surgery. Anteroposterior view (left) and lateral view (right).

**Figure 5:**
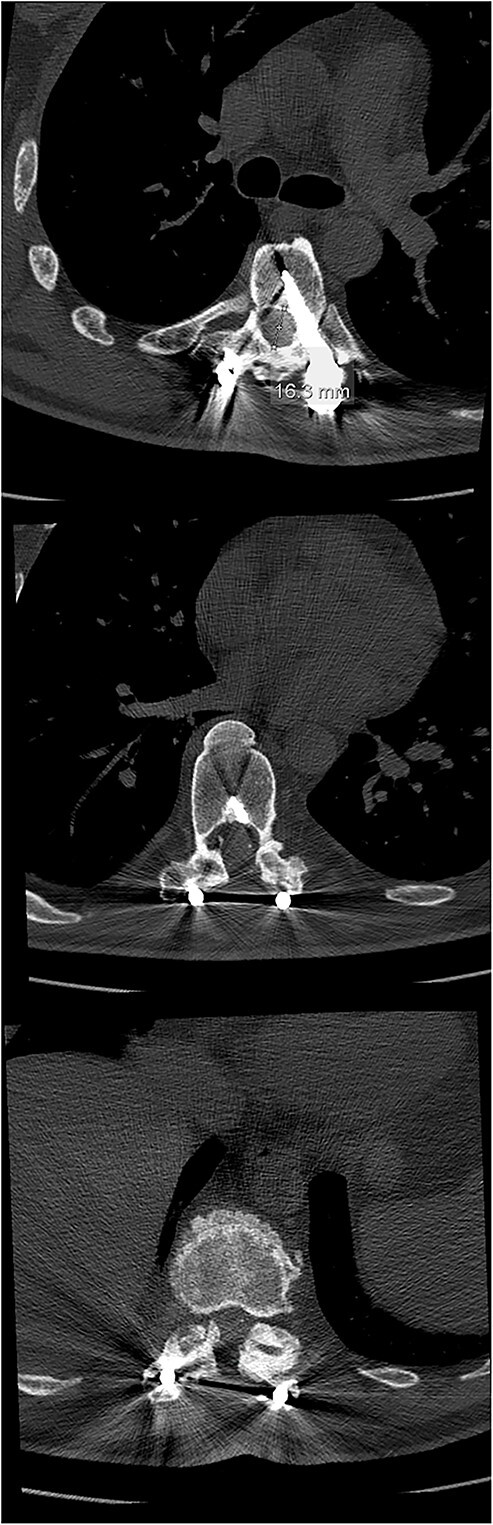
Final follow-up CT scan (axial views), showing adequate width of the spinal canal at Th6 (above), Th9 (middle) and Th11 (below).

Six weeks after surgery he was transferred to a tertiary rehabilitation center, ambulating using a walker, but remained wheelchair-bound for longer distances. The postoperative pain was manageable with non-opioids. During the regular follow-ups, the paraplegia with severe spasticity persisted, without significant pain. A subsequent insertion of the intrathecal baclofen pump in a tertiary rehabilitation facility enabled the patient to manage the muscle spasms in his lower limbs, particularly during transfers from the wheelchair to bed and vice versa. The patient was able to urinate spontaneously with minimal urine retention, and no need for self-catheterisation, reporting satisfaction with the outcome of the surgery and rehabilitation, which enabled him a return to his workplace and retain a significant level of independence in everyday activities. On the final follow-up imaging, 4 years after the surgery, no significant deformity progression and no evidence of neurologic impairment were observed (Figs 4 and 5).

## DISCUSSION

The OLF is considered one of the most significant causes of thoracic myelopathy, particularly in eastern Asia, where its prevalence has been the highest [1, 3, 7–9]. On the contrary, reports on the European population, like in this presented case, are scarce [7].

Multiple factors have been associated with the development of the OLF, including age, gender, genetic variance, high BMI and conditions, such as hypoparathyroidism, diabetes mellitus and bone tuberculosis [1, 2, 4, 5]. However, the definite pathophysiology of the OLF has not been fully understood yet [4, 6]. The latter dilemma is also present in our case, where, despite our patient being male and associated with multiple neurological conditions, related to his spine and central nervous system, he was relatively young, without history of metabolic disorders.

In up to 51% of patients in need of decompression surgery due to thoracic myelopathy, the presence of the OLF was noted, whereby the prominent clinical feature in these patients was the deficit in vibratory sensation and proprioception on account of the compressed dorsal spinal cord [2, 5, 8]. The numbness was also present in our patient, along with significant pain and acute aggravation of paraplegia. The most prevalent location of OLF is the lower thoracic spine with the highest peak being at Th10-12 (and the second highest at Th3-5, respectively), whereby the ossification of the other spinal ligaments can accompany OLF [1, 3, 9]. This was also observed in our case, where the most significant spinal stenosis was also observed in lower thoracic segments (namely Th6, Th9 and Th11).

Computed tomography (CT) is considered the most useful diagnostic imaging modality in identifying the ossification of the spinal ligaments, including the OLF [3, 8]. In severe cases, the ossification can also include the dorsal area of the dura, making the surgical treatment of the OLF very challenging, with a high risk of a dural compromise and leakage of the cerebrospinal fluid [2, 5, 8, 10]. However, this was not the case with our patient.

Spinal deformities, with an associated alteration in spinal biomechanics, have also been associated with OLF, especially thoracic kyphosis [1, 11, 12]. Thus, the presence of kyphoscoliosis in our patient could imply the contribution of the deformity to the development of the OLF [1, 11, 12]. The lower thoracic spine acts as a transitional area regarding spinal curvatures; therefore, it may be subjected to concomitant repetitive exposure of the ligamentum flavum to the excessive tensile force, leading to hypertrophy and ossification of the ligament [1, 2, 4, 6]. However, it is not clearly distinguished whether the course of the deformity progression in our patient was natural or amplified due to two previous laminectomies due to intramedullary cavernous hemangioma removal [11].

## Data Availability

This manuscript presents a case report and therefore the data are not available publicly or upon request to protect the privacy and identity of the patient.
